# (*E*)-1-(4-Bromo­phen­yl)-3-(2-chloro­phen­yl)prop-2-en-1-one

**DOI:** 10.1107/S1600536808020795

**Published:** 2008-07-12

**Authors:** Hoong-Kun Fun, P. S. Patil, S. M. Dharmaprakash, Suchada Chantrapromma

**Affiliations:** a X-ray Crystallography Unit, School of Physics, Universiti Sains Malaysia, 11800 USM, Penang, Malaysia; b Department of Studies in Physics, Mangalore University, Mangalagangotri, Mangalore 574 199, India; c Crystal Materials Research Unit, Department of Chemistry, Faculty of Science, Prince of Songkla University, Hat-Yai, Songkhla 90112, Thailand

## Abstract

The structure of the title compound, C_15_H_10_BrClO, comprises two substituted benzene rings bridged by a prop-2-en-1-one group and exists in an *E* configuration about the C=N double bond. The dihedral angle formed between the 4-bromo­phenyl and 2-chloro­phenyl rings is 23.77 (18)°. In the crystal structure, the mol­ecules are linked by weak C—H⋯O inter­actions, forming a supra­molecular zigzag chain. Intramolecular C—H⋯Cl and C—H⋯O hydrogen bonds are also present.

## Related literature

For related literature on hydrogen-bond motifs, see: Bernstein *et al.* (1995 ▶). For related structures, see: Patil *et al.* (2007 ▶); Moorthi *et al.* (2005 ▶). For applications of chalcones, see: Gu *et al.* (2008 ▶); Mishra *et al.* (2008 ▶); Nel *et al.* (1998 ▶); Patil & Dharmaprakash (2008 ▶); Wang *et al.* (2004 ▶).
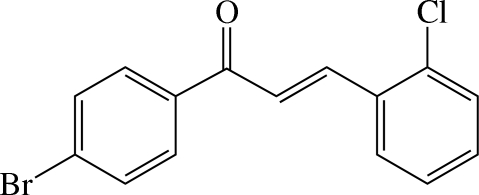


## Experimental

### 

#### Crystal data

C_15_H_10_BrClO
*M* *_r_* = 321.59
Orthorhombic, 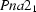

*a* = 27.8720 (6) Å
*b* = 3.9235 (1) Å
*c* = 11.6408 (2) Å
*V* = 1272.99 (5) Å^3^
*Z* = 4
Mo *K*α radiation
μ = 3.42 mm^−1^
*T* = 100.0 (1) K
0.33 × 0.18 × 0.09 mm

#### Data collection

Bruker SMART APEX2 CCD area-detector diffractometer
Absorption correction: multi-scan (*SADABS*; Bruker, 2005 ▶) *T*_min_ = 0.392, *T*_max_ = 0.736
9658 measured reflections
3495 independent reflections
2938 reflections with *I* > 2σ(*I*)
*R*_int_ = 0.044

#### Refinement

*R*[*F* ^2^ > 2σ(*F* ^2^)] = 0.033
*wR*(*F* ^2^) = 0.086
*S* = 1.03
3495 reflections
163 parameters
1 restraint
H-atom parameters constrained
Δρ_max_ = 0.41 e Å^−3^
Δρ_min_ = −0.44 e Å^−3^
Absolute structure: Flack (1983 ▶), 1545 Friedel pairs
Flack parameter: 0.011 (12)

### 

Data collection: *APEX2* (Bruker, 2005 ▶); cell refinement: *APEX2*; data reduction: *SAINT* (Bruker, 2005 ▶); program(s) used to solve structure: *SHELXTL* (Sheldrick, 2008 ▶); program(s) used to refine structure: *SHELXTL*; molecular graphics: *SHELXTL* software used to prepare material for publication: *SHELXTL* and *PLATON* (Spek, 2003 ▶).

## Supplementary Material

Crystal structure: contains datablocks global, I. DOI: 10.1107/S1600536808020795/tk2281sup1.cif

Structure factors: contains datablocks I. DOI: 10.1107/S1600536808020795/tk2281Isup2.hkl

Additional supplementary materials:  crystallographic information; 3D view; checkCIF report

## Figures and Tables

**Table 1:** Hydrogen-bond geometry (Å, °)

*D*—H⋯*A*	*D*—H	H⋯*A*	*D*⋯*A*	*D*—H⋯*A*
C1—H1*A*⋯O1^i^	0.93	2.53	3.191 (4)	128
C9—H9*A*⋯Cl1	0.93	2.61	3.064 (4)	111
C9—H9*A*⋯O1	0.93	2.41	2.765 (5)	102

Symmetry code: (i) 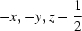.

**Fig. 1.**
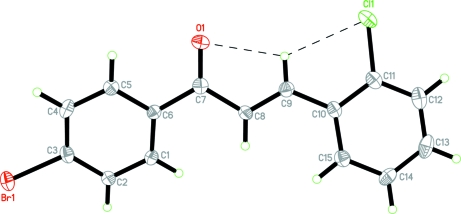
The molecular structure of (I), showing 50% probability displacement ellipsoids and the atom-numbering scheme. The dashed lines represent intramolecular C—H···O and C—H···Cl interactions.

**Fig. 2.**
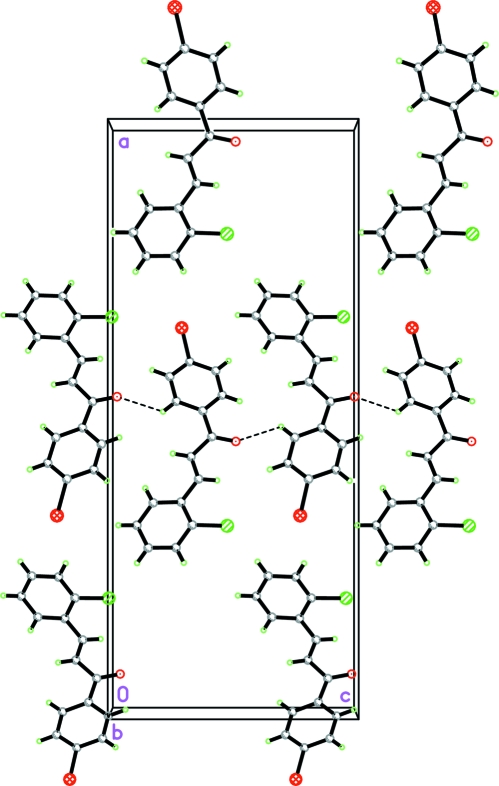
A view down the b-axis of the crystal packing in (I), highlighting a supramolecular molecular chain aligned along the c axis. The C-H···O interactions are shown as dashed lines.

## supplementary crystallographic information

### Comment

Chalcone and its derivatives have a wide range of applications ranging from bioactivities (Mishra *et al.*, 2008; Nel *et al.*, 1998) to materials with non-linear optical (NLO) properties (Gu *et al.*, 2008 & Moorthi *et al.*, 2005). As part of our continuing interest in the latter application (Patil & Dharmaprakash, 2008), the synthesis and structure of the title compound (I, Fig. 1) is described herein.The non-centrosymmetric crystal of the title compound should exhibit 2nd-order NLO properties.

The structure of (I) comprises two six-membered rings bridged by a pro-2-en-1-one moiety. The molecule exists in the *E* conformation with respect to the C8=C9 double bond [1.328 (5) Å]. The molecule is not planar as seen in the dihedral angle of 23.77 (18)° formed between the 4-bromophenyl and 2-chlorophenyl rings. Further, the mean plane through the O1, C6, C7 & C8 atoms forms angles, respectively, of 13.2 (2)° and 11.0 (2)° with the planes of 4-bromophenyl and 2-chlorophenyl rings. Weak C9–H9A···O1 and C9—H9A···Cl1 intramolecular interactions (Fig. 1 & Table 1) generate S(5) ring motifs (Bernstein *et al.*, 1995). The derived bond distances and angles are comparable with those determined in the closely related structures (e.g. Patil *et al.*, 2007 & Sathiya Moorthi *et al.*, 2005).

In the crystal packing (Fig. 2), the molecules are linked into a supramolecular chain via C-H···O interactions aligned along the c-direction, Table 1.

### Experimental

Compound (I) was synthesized by the condensation of 2-chlorobenzaldehyde (0.01 mol, 1.49 g) with 4-bromoacetophenone (0.01 mol, 1.99 g) in methanol (60 ml) in the presence of a catalytic amount of sodium hydroxide solution (5 ml, 20%). After stirring for 2 h, the contents of the flask were poured into ice-cold water (500 ml) and left to stand for 5 h. The resulting crude solid was filtered and dried. Single crystals were obtained by recrystallization from an acetone solution of (I).

### Refinement

All H atoms were in the riding model approximation with C—H = 0.93 Å, and with *U*_iso_ = 1.2*U*_eq_(C).

### Crystal data

**Table:**

C_15_H_10_BrClO	*F*_000_ = 640
*M**_r_* = 321.59	*D*_x_ = 1.678 Mg m^−^^3^
Orthorhombic, *P**n**a*2_1_	Mo *K*α radiation λ = 0.71073 Å
Hall symbol: P 2c -2n	Cell parameters from 3495 reflections
*a* = 27.8720 (6) Å	θ = 1.5–30.0º
*b* = 3.9235 (1) Å	µ = 3.42 mm^−^^1^
*c* = 11.6408 (2) Å	*T* = 100.0 (1) K
*V* = 1272.99 (5) Å^3^	Block, colorless
*Z* = 4	0.33 × 0.18 × 0.09 mm

### Data collection

**Table:**

Bruker SMART APEX2 CCD area-detector diffractometer	3495 independent reflections
Radiation source: fine-focus sealed tube	2938 reflections with *I* > 2σ(*I*)
Monochromator: graphite	*R*_int_ = 0.044
Detector resolution: 8.33 pixels mm^-1^	θ_max_ = 30.0º
*T* = 100.0(1) K	θ_min_ = 1.5º
ω scans	*h* = −36→39
Absorption correction: multi-scan(SADABS; Bruker, 2005)	*k* = −5→3
*T*_min_ = 0.392, *T*_max_ = 0.736	*l* = −16→16
9658 measured reflections	

### Refinement

**Table:**

Refinement on *F*^2^	Hydrogen site location: inferred from neighbouring sites
Least-squares matrix: full	H-atom parameters constrained
*R*[*F*^2^ > 2σ(*F*^2^)] = 0.033	*w* = 1/[σ^2^(*F*_o_^2^) + 1.3265*P*] where *P* = (*F*_o_^2^ + 2*F*_c_^2^)/3
*wR*(*F*^2^) = 0.086	(Δ/σ)_max_ = 0.001
*S* = 1.03	Δρ_max_ = 0.41 e Å^−^^3^
3495 reflections	Δρ_min_ = −0.44 e Å^−^^3^
163 parameters	Extinction correction: none
1 restraint	Absolute structure: Flack (1983), 1545 Friedel pairs
Primary atom site location: structure-invariant direct methods	Flack parameter: 0.011 (12)
Secondary atom site location: difference Fourier map	

### Special details

**Table:**

Experimental. The low-temperature data was collected with the Oxford Cyrosystem Cobra low-temperature attachment.
Geometry. All e.s.d.'s (except the e.s.d. in the dihedral angle between two l.s. planes) are estimated using the full covariance matrix. The cell e.s.d.'s are taken into account individually in the estimation of e.s.d.'s in distances, angles and torsion angles; correlations between e.s.d.'s in cell parameters are only used when they are defined by crystal symmetry. An approximate (isotropic) treatment of cell e.s.d.'s is used for estimating e.s.d.'s involving l.s. planes.
Refinement. Refinement of *F*^2^ against ALL reflections. The weighted *R*-factor *wR* and goodness of fit *S* are based on *F*^2^, conventional *R*-factors *R* are based on *F*, with *F* set to zero for negative *F*^2^. The threshold expression of *F*^2^ > σ(*F*^2^) is used only for calculating *R*-factors(gt) *etc*. and is not relevant to the choice of reflections for refinement. *R*-factors based on *F*^2^ are statistically about twice as large as those based on *F*, and *R*- factors based on ALL data will be even larger.

### Fractional atomic coordinates and isotropic or equivalent isotropic displacement parameters (Å^2^)

**Table:**

	*x*	*y*	*z*	*U*_iso_*/*U*_eq_	
Br1	0.165695 (11)	0.71499 (8)	0.27469 (5)	0.02346 (10)	
Cl1	−0.18444 (4)	−0.5643 (3)	0.47411 (9)	0.0264 (2)	
O1	−0.03953 (10)	0.0955 (8)	0.5096 (2)	0.0254 (6)	
C1	0.03194 (13)	0.2560 (9)	0.2561 (3)	0.0187 (8)	
H1A	0.0132	0.1502	0.2004	0.022*	
C2	0.07645 (13)	0.3879 (11)	0.2266 (3)	0.0192 (8)	
H2A	0.0878	0.3720	0.1517	0.023*	
C3	0.10349 (14)	0.5426 (9)	0.3106 (3)	0.0192 (8)	
C4	0.08725 (13)	0.5741 (10)	0.4230 (3)	0.0202 (8)	
H4A	0.1059	0.6831	0.4782	0.024*	
C5	0.04319 (13)	0.4411 (9)	0.4510 (3)	0.0177 (8)	
H5A	0.0321	0.4585	0.5261	0.021*	
C6	0.01502 (13)	0.2810 (9)	0.3686 (3)	0.0156 (7)	
C7	−0.03108 (13)	0.1245 (10)	0.4073 (3)	0.0180 (8)	
C8	−0.06613 (13)	0.0089 (10)	0.3202 (3)	0.0185 (8)	
H8A	−0.0610	0.0543	0.2428	0.022*	
C9	−0.10501 (14)	−0.1603 (10)	0.3534 (3)	0.0192 (8)	
H9A	−0.1074	−0.2079	0.4315	0.023*	
C10	−0.14477 (11)	−0.2809 (8)	0.2809 (5)	0.0180 (6)	
C11	−0.18352 (14)	−0.4627 (10)	0.3288 (3)	0.0206 (8)	
C12	−0.22195 (13)	−0.5699 (9)	0.2625 (4)	0.0246 (8)	
H12A	−0.2470	−0.6907	0.2960	0.030*	
C13	−0.22293 (15)	−0.4970 (11)	0.1464 (4)	0.0276 (9)	
H13A	−0.2488	−0.5662	0.1017	0.033*	
C14	−0.18509 (15)	−0.3199 (11)	0.0968 (3)	0.0239 (8)	
H14A	−0.1856	−0.2719	0.0185	0.029*	
C15	−0.14652 (15)	−0.2140 (10)	0.1632 (3)	0.0213 (8)	
H15A	−0.1214	−0.0963	0.1287	0.026*	

### Atomic displacement parameters (Å^2^)

**Table:**

	*U*^11^	*U*^22^	*U*^33^	*U*^12^	*U*^13^	*U*^23^
Br1	0.01631 (16)	0.02108 (18)	0.03298 (17)	−0.00286 (13)	0.0010 (2)	0.0002 (3)
Cl1	0.0257 (5)	0.0253 (5)	0.0282 (4)	−0.0021 (4)	0.0095 (4)	0.0033 (4)
O1	0.0260 (16)	0.0329 (18)	0.0171 (13)	−0.0047 (13)	0.0038 (11)	0.0008 (11)
C1	0.0159 (16)	0.0214 (19)	0.019 (2)	−0.0002 (13)	−0.0025 (13)	0.0006 (14)
C2	0.0177 (19)	0.021 (2)	0.0187 (16)	−0.0023 (15)	−0.0003 (14)	0.0018 (15)
C3	0.0170 (18)	0.0126 (19)	0.0278 (19)	0.0004 (14)	−0.0001 (14)	0.0023 (14)
C4	0.0186 (19)	0.017 (2)	0.0248 (19)	0.0025 (15)	−0.0068 (15)	−0.0046 (15)
C5	0.0180 (18)	0.017 (2)	0.0180 (17)	0.0023 (14)	0.0011 (13)	−0.0021 (14)
C6	0.0138 (17)	0.0161 (19)	0.0169 (16)	0.0025 (14)	−0.0010 (13)	0.0003 (13)
C7	0.0176 (18)	0.015 (2)	0.0218 (17)	0.0042 (14)	−0.0011 (14)	0.0011 (14)
C8	0.0153 (18)	0.022 (2)	0.0178 (16)	−0.0010 (15)	0.0015 (14)	0.0020 (14)
C9	0.018 (2)	0.020 (2)	0.0195 (16)	0.0021 (15)	−0.0003 (14)	0.0000 (14)
C10	0.0150 (14)	0.0143 (15)	0.0248 (15)	0.0030 (12)	0.001 (2)	0.0027 (19)
C11	0.0184 (19)	0.015 (2)	0.0280 (19)	0.0060 (15)	0.0042 (15)	0.0010 (15)
C12	0.0177 (17)	0.0158 (18)	0.040 (2)	0.0019 (13)	0.0016 (18)	−0.004 (2)
C13	0.020 (2)	0.022 (2)	0.041 (2)	0.0077 (17)	−0.0090 (18)	−0.0105 (18)
C14	0.025 (2)	0.025 (2)	0.0220 (19)	0.0065 (17)	−0.0067 (16)	−0.0013 (16)
C15	0.021 (2)	0.017 (2)	0.0257 (19)	−0.0003 (15)	0.0010 (15)	0.0019 (15)

### Geometric parameters (Å, °)

**Table:**

Br1—C3	1.907 (4)	C8—C9	1.328 (5)
Cl1—C11	1.738 (4)	C8—H8A	0.9300
O1—C7	1.219 (4)	C9—C10	1.472 (6)
C1—C2	1.387 (5)	C9—H9A	0.9300
C1—C6	1.396 (5)	C10—C15	1.395 (7)
C1—H1A	0.9300	C10—C11	1.409 (5)
C2—C3	1.376 (5)	C11—C12	1.386 (6)
C2—H2A	0.9300	C12—C13	1.381 (7)
C3—C4	1.390 (5)	C12—H12A	0.9300
C4—C5	1.373 (5)	C13—C14	1.389 (6)
C4—H4A	0.9300	C13—H13A	0.9300
C5—C6	1.389 (5)	C14—C15	1.388 (6)
C5—H5A	0.9300	C14—H14A	0.9300
C6—C7	1.493 (5)	C15—H15A	0.9300
C7—C8	1.479 (5)		
			
C2—C1—C6	120.5 (3)	C7—C8—H8A	120.2
C2—C1—H1A	119.7	C8—C9—C10	127.4 (4)
C6—C1—H1A	119.7	C8—C9—H9A	116.3
C3—C2—C1	118.6 (3)	C10—C9—H9A	116.3
C3—C2—H2A	120.7	C15—C10—C11	117.2 (4)
C1—C2—H2A	120.7	C15—C10—C9	122.0 (3)
C2—C3—C4	122.0 (4)	C11—C10—C9	120.8 (5)
C2—C3—Br1	119.9 (3)	C12—C11—C10	121.7 (4)
C4—C3—Br1	118.1 (3)	C12—C11—Cl1	117.5 (3)
C5—C4—C3	118.7 (3)	C10—C11—Cl1	120.8 (3)
C5—C4—H4A	120.6	C13—C12—C11	119.8 (4)
C3—C4—H4A	120.6	C13—C12—H12A	120.1
C4—C5—C6	120.9 (3)	C11—C12—H12A	120.1
C4—C5—H5A	119.6	C12—C13—C14	119.7 (4)
C6—C5—H5A	119.6	C12—C13—H13A	120.1
C5—C6—C1	119.2 (3)	C14—C13—H13A	120.1
C5—C6—C7	117.7 (3)	C15—C14—C13	120.4 (4)
C1—C6—C7	123.0 (3)	C15—C14—H14A	119.8
O1—C7—C8	120.9 (4)	C13—C14—H14A	119.8
O1—C7—C6	119.9 (3)	C14—C15—C10	121.2 (4)
C8—C7—C6	119.2 (3)	C14—C15—H15A	119.4
C9—C8—C7	119.5 (3)	C10—C15—H15A	119.4
C9—C8—H8A	120.2		
			
C6—C1—C2—C3	0.1 (6)	C6—C7—C8—C9	173.5 (4)
C1—C2—C3—C4	−0.9 (6)	C7—C8—C9—C10	176.9 (3)
C1—C2—C3—Br1	178.1 (3)	C8—C9—C10—C15	−2.7 (6)
C2—C3—C4—C5	1.1 (6)	C8—C9—C10—C11	179.0 (4)
Br1—C3—C4—C5	−177.9 (3)	C15—C10—C11—C12	−0.3 (5)
C3—C4—C5—C6	−0.7 (6)	C9—C10—C11—C12	178.1 (3)
C4—C5—C6—C1	0.0 (6)	C15—C10—C11—Cl1	179.1 (3)
C4—C5—C6—C7	176.4 (3)	C9—C10—C11—Cl1	−2.5 (5)
C2—C1—C6—C5	0.3 (6)	C10—C11—C12—C13	−0.4 (6)
C2—C1—C6—C7	−175.9 (4)	Cl1—C11—C12—C13	−179.8 (3)
C5—C6—C7—O1	−10.8 (5)	C11—C12—C13—C14	0.7 (6)
C1—C6—C7—O1	165.5 (4)	C12—C13—C14—C15	−0.4 (6)
C5—C6—C7—C8	168.8 (3)	C13—C14—C15—C10	−0.2 (6)
C1—C6—C7—C8	−15.0 (5)	C11—C10—C15—C14	0.6 (5)
O1—C7—C8—C9	−7.0 (6)	C9—C10—C15—C14	−177.8 (4)

### Hydrogen-bond geometry (Å, °)

**Table:**

*D*—H···*A*	*D*—H	H···*A*	*D*···*A*	*D*—H···*A*
C1—H1A···O1^i^	0.93	2.53	3.191 (4)	128
C9—H9A···Cl1	0.93	2.61	3.064 (4)	111
C9—H9A···O1	0.93	2.41	2.765 (5)	102

Symmetry codes: (i) −*x*, −*y*, *z*−1/2.
